# Adjustment of sex allocation to co‐foundress number and kinship under local mate competition: An inclusive‐fitness analysis

**DOI:** 10.1111/jeb.13719

**Published:** 2020-11-18

**Authors:** Andy Gardner, Ian C. W. Hardy

**Affiliations:** ^1^ School of Biology University of St Andrews St Andrews UK; ^2^ School of Biosciences University of Nottingham Loughborough UK

**Keywords:** foundress kinship, local mate competition, parasitoid, sex ratio

## Abstract

Hamilton's theory of local mate competition (LMC) describes how competition between male relatives for mating opportunities favours a female‐biased parental investment. LMC theory has been extended in many ways to explore a range of genetic and life‐history influences on sex allocation strategies, including showing that increasing genetic homogeneity within mating groups should favour greater female bias. However, there has been no quantitative theoretical prediction as to how females should facultatively adjust their sex allocation in response to co‐foundress number and kinship. This shortfall has been highlighted recently by the finding that sex ratios produced by sub‐social parasitoid wasps in the family Bethylidae are affected by the number of co‐foundresses and by whether these are sisters or unrelated females. Here we close this gap in LMC theory by taking an inclusive‐fitness approach to derive explicit theoretical predictions for this scenario. We find that, in line with the recent empirical results, females should adopt a more female‐biased sex allocation when their co‐foundresses are less numerous and are their sisters. Our model appears to predict somewhat more female bias than is observed empirically; we discuss a number of possible model extensions that would improve realism and that would be expected to result in a closer quantitative fit with experimental data.

## Graphical abstract

Sub‐social parasitoids are predicted to adjust their sex allocation behaviour when co‐foundresses are sisters.

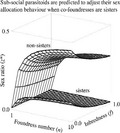

## 1 INTRODUCTION

The theory of sex allocation, which concerns the trade‐off between female vs male reproductive effort, has been described as the ‘jewel in the crown of evolutionary ecology’ (West & Herre, 2002), and it provides among the best evidence of the precision of Darwinian adaptation in the natural world (West, 2009). Perhaps its most productive application has been to scenarios in which mating groups comprise genetic relatives, such that wasteful competition among males induces parents to decrease their investment into sons. Hamilton (1967) derived an unbeatable sex allocation strategy for such ‘local mate competition’ (LMC) under a diplo‐diploid mode of inheritance. This showed that a mother who is one of *n* unrelated females contributing offspring to a mating group should make a proportional investment of (*n*–1)/(2*n*) into sons, such that she should invest nearly all of her reproductive effort into daughters (producing just enough sons to fertilize them) if she is the only mother present, and make a nearly equal investment into daughters and sons if she is one of very many females contributing offspring to the mating group, recovering Fisher’s (1930) classic result for large, panmictic populations.

Hamilton’s (1967) analysis has subsequently been extended in a number of ways (reviewed by West, 2009), including to allow for a haplo‐diploid mode of inheritance (Hamilton, 1979) and kinship among co‐foundresses (Bulmer, 1986; Courteau & Lessard, 2000; Frank, 1985, 1986a, b, 1998; Gardner et al., 2009; Rodrigues & Gardner, 2015; Taylor, 1988; Taylor & Crespi, 1994). A very general result has been provided by Frank (1985, 1986a) who showed that the unbeatable sex allocation under LMC is given by the product of three terms: ½, reflecting the rarer‐sex effect of Fisher (1930); *R*, capturing any asymmetry in the inclusive‐fitness valuation of a son vs a daughter (such that *R* < 1 if daughters are valued more, *R* > 1 if sons are valued more and *R* = 1 if both are valued equally); and *P_d_*_(_ *_a_*_)_ *_t_*, representing Wright’s (1969) coefficient of panmixia, and hence, the degree to which mating groups are genetically heterogeneous. Frank (1985) has highlighted that the coefficient of panmixia may vary between groups and hence that females might be favoured to adjust their sex allocation according to their local assessment of both number and kinship of co‐foundresses, though no explicit, quantitative results have so far been derived for this particular scenario.

This gap in the theoretical development of LMC has recently been highlighted by an empirical study of sex allocation in *Goniozus* wasps (Abdi et al., 2020); this found that collective brood sex ratios are affected by both the number of foundresses and kinship among co‐foundresses. Briefly, *Goniozus* species are haplo‐diploid parasitoids which have long been known to exhibit female‐biased sex ratios and comply broadly with both the assumptions and predictions of LMC theory (Green et al., 1982; Hamilton, 1967, 1979; Hardy & Cook, 1995; Hardy et al., 1999; Khidr et al., 2013). Due to aggressive resource competition between females and subsequent brood care (sub‐social reproduction), the number of foundresses contributing offspring to a mating group is thought to be typically just one. Yet adult females are able to discriminate kinship and may tolerate each other's presence when relatedness is higher and also when host resources are less limiting (Abdi et al., 2020; Lizé et al., 2012). When experimentally induced to reproduce in multi‐foundress groups, the sex ratios of broods produced by sibling females were similar to sex ratios produced by single foundresses (proportion of offspring that were male ≈ 0.10) whereas nonsibling foundresses produced sex ratios were much higher (≈ 0.40; Abdi et al., 2020).

Here we close the theory gap by deriving explicit theoretical predictions for scenarios in which a female may facultatively adjust her sex allocation according to the number of her co‐foundresses and whether they are her sisters or are unrelated females. We take an inclusive‐fitness approach (Hamilton, 1964), showing that the female's unbeatable sex ratio depends not only on these two factors, but also on the average degree of inbredness across the whole population. We provide solutions for both diplo‐diploid and haplo‐diploid modes of genetic inheritance and find an improved fit between sex ratio predictions for haplo‐diploidy and the *Goniozus* sex ratios observed by Abdi et al. (2020). Despite this improvement, our model does appear to predict somewhat more female bias than is observed empirically. Accordingly, we discuss a number of possible model extensions—including partial male dispersal and local resource competition—that would further improve realism and that would be expected to result in a closer quantitative fit with the experimental data.

## 2 MODEL AND RESULTS

We consider a foundress group in which there are *n* females each making an equal contribution of offspring to a mating group, with each female by default adopting a sex allocation strategy *z* such that she contributes *Nz* sons and *N*(1–*z*) daughters, where *N* is a large number. Their offspring then mate at random among each other, with each female mating once and each male potentially mating a large number of times. Following mating, the males die, and the mated females disperse to form new foundress groups with other females drawn at random from the entire population. We assume that these new foundress groups almost always comprise unrelated females, but we do allow for a nonzero probability that co‐foundresses are sisters in order to investigate how females are favoured to behave in such circumstances.

To determine unbeatable sex allocation behaviour, we focus attention on one of the *n* foundresses and consider the inclusive‐fitness consequences of her adopting an alternative sex allocation strategy *z* + δ, such that she instead contributes *N*(*z* + δ) sons and *N*(1–*z*–δ) daughters to the mating group (full details are given in Box 1).

Box 1 **Inclusive‐fitness derivation**

**General**

The focal female produces *N*_d_ = *N*(1–*z*–δ) daughters and *N*_s_ = *N*(*z* + δ) sons, and the *n*–1 other females in her foundress group collectively produce *N*_f_ = (*n*−1)*N*(1–*z*) daughters and *N*_m_ = (*n*−1)*Nz* sons. Accordingly, the total inclusive‐fitness (Hamilton, 1964) value the focal female places upon the mating group is.

(B1.1) $$H=N_{d}p_{d}v_{f}+N_{f}p_{f}v_{f}+\left(N_{d}+N_{f}\right)\frac{N_{s}}{N_{s}+N_{m}}p_{s}v_{m}+\left(N_{d}+N_{f}\right)\frac{N_{m}}{N_{s}+N_{m}}p_{m}v_{m}$$

where *p*_d_ is her consanguinity (i.e. probability of identity by descent; Bulmer, 1994) to her daughters, *p*_s_ is her consanguinity to her sons, *p*_f_ is her consanguinity to the daughters of her co‐foundresses, *p*_m_ is her consanguinity to the sons of her co‐foundresses, *v*_f_ is the reproductive value (Bulmer, 1994; Fisher, 1930; Hamilton, 1972) of a mated female's eggs and *v*_m_ is the reproductive value of the sperm that fertilize a mated female's eggs. The inclusive‐fitness effect of a small deviation δ in the focal female's sex allocation strategy is ∆*H* = (∂*H*/∂δ|_δ=0_)δ and, accordingly, the unbeatable sex allocation strategy (Hamilton, 1967) satisfies ∆*H*|*_z_*_=_ _z__*_ = 0, which yields.

(B1.2) $$z^{*}=\frac{\left(n‐1\right)\left(p_{s}‐p_{m}\right)v_{m}}{n\left(p_{d}v_{f}+p_{s}v_{m}\right)}.$$

The right‐hand side of equation (B1.2) is equivalent to the expression ½ *R P_d_*_(_ *_a_*_)_ *_t_* derived by Frank (1985; see also Hamilton, 1979, Frank, 1986a), using a different, lengthier and less‐accessible approach, where the ½ term captures the rarer‐sex effect, the *R* = (2 *p*_s_ *v*_m_)/(*p*_d_ *v*_f_ + *p*_s_ *v*_m_) term is a coefficient of inheritance asymmetry between the sexes and the *P_d_*_(_ *_a_*_)_ *_t_* = ((*n*−1)/*n*)(*p*_s_–*p*_m_)/*p*_s_ term is an index of panmixia as assessed by the focal female conditional upon the information she has available to her—potentially including number and relatedness of co‐foundresses. Note that, as *p*_m_ is the only determinant of z* that is affected if a female conditions her sex allocation upon whether her co‐foundresses are or are not her sisters, and since z* is linear in *p*_m_, it is evident that kin discrimination is not expected to affect the overall sex ratio of the population compared with a scenario in which females cannot discriminate kin (*cf*. Faria & Gardner, 2020). Nor would any ability to detect variation in genetic relatedness among sisters affect the average sex allocation employed in response to sister co‐foundresses (*cf*. Faria & Gardner, 2020). Moreover, as *n* is the only determinant of z* that is affected if a female conditions her sex allocation upon the number of her co‐foundresses, and since z* is a concave function of *n*, it is evident that such conditionality is expected to reduce the overall sex ratio of the population compared with a scenario in which females cannot adjust their sex allocation in response to co‐foundress number (*cf*. Faria & Gardner, 2020).

**Diplo‐diploidy**

Under diplo‐diploidy, *p*_d_ = *p*_s_ = (1 + 3*f*)/4, where *f* is the consanguinity of the focal female's parents and hence describes her ‘inbredness’ (Frank, 1985, 1986a), and *v*_f_ = *v*_m_ (Bulmer, 1994; Fisher, 1930). Accordingly, if the focal female is unrelated to her co‐foundresses, such that *p*_m_ = 0, then her unbeatable sex allocation strategy is given by Equation (1) of the main text.

If instead the focal female's co‐foundresses are her sisters, then *p*_m_ = (1 + 7*f*)/8 and her unbeatable sex allocation strategy is given by Equation (2) of the main text. Note that this equation depends on the focal female's inbredness, which depends on the frequency of sib‐matings (and hence the size of foundress groups) among her ancestors. If the number of foundresses is *n* in all foundress groups, not just in the focal female's foundress group, then inbredness may be expressed as *f* = 1/(4*n*−3), such that the unbeatable sex allocation strategy is *z*_D,S_* = (*n*–1)^2^/(4*n* ^2^). If the number of foundresses is variable, then *f* = 1/(4*ν*–3) and *z*_D,S_* = ((*n*–1)(ν–1))/(4*n*ν), where ν is the harmonic mean foundress number (specifically, taken across all females, it is the harmonic mean of the number of foundresses in their mothers’ foundress groups; *cf*. Frank, 1985).

**Haplo‐diploidy**

Under haplo‐diploidy, *p*_d_ = (1 + 3*f*)/4, *p*_s_ = (1 + *f*)/2 and *v*_f_ = 2*v*_m_ (Bulmer, 1994; Hamilton, 1972). Accordingly, if the focal female is unrelated to her co‐foundresses, such that *p*_m_ = 0, then her unbeatable sex allocation strategy is given by Equation (3) of the main text. If the number of foundresses contributing to different mating groups is constant, then *f* = 1/(4*n*‐3) and *z*_H,U_* = ((*n*–1)(2*n* – 1))/(*n*(4*n*–1)). If the number of foundresses is variable, then *f* = 1/(4*ν*–3) and *z*_H,U_* = ((*n*–1)(2*ν*–1))/(*n*(4*ν*–1)).

Finally, if the focal female's co‐foundresses are her sisters, then *p*_m_ = (3 + 5*f*)/8 and her unbeatable sex allocation strategy is given by Equation (4) of the main text. If the number of foundresses contributing to different mating groups is constant, then *f* = 1/(4*n*−3) and *z*_H,S_* = (*n*–1)^2^/(2*n*(4*n*–1)), and if the number of foundresses is variable, then *f* = 1/(4*ν*–3) and *z*_H,S_* = ((*n*–1)(*ν*–1))/(2*n*(4*ν*–1)).

**FIGURE 1:**
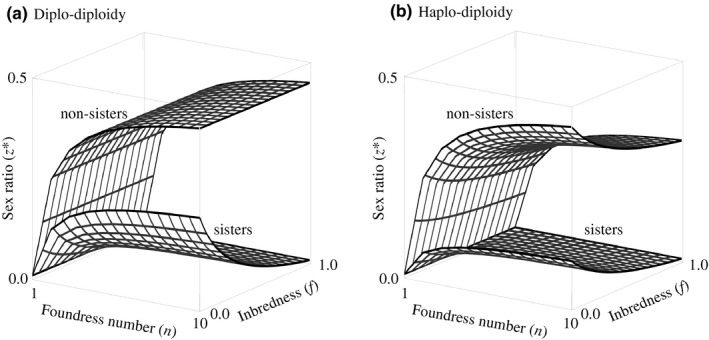
Predicted sex ratios in groups of sister or nonsister foundresses, according to foundress number and inbredness. Panel (a) shows optima for diplo‐diploid inheritance, and panel (b) shows optima for haplo‐diploid inheritance

First, we consider a diplo‐diploid (D) mode of inheritance. Here, we find that the unbeatable sex allocation for a female whose group comprises herself and *n*−1 unrelated (U) other females is given by.

(1) $$z_{D,U}^{*}=\frac{n‐1}{2n}$$

which is exactly the result given by Hamilton (1967). This result is illustrated by the upper surface in Figure 1a. Note that Equation (1) holds irrespective of whether the number of foundresses is constant or variable across groups and depends only on the number of foundresses present in the female's own group (see Box 1 for details). In contrast, we find that the unbeatable sex allocation for a female whose group comprises herself and *n*‐1 of her sisters (S) is given by.

(2) $$z_{D,S}^{*}=\frac{\left(n‐1\right)\left(1‐f\right)}{4n\left(1+3f\right)}$$

where *f* describes the ‘inbredness’ of females, that is the average consanguinity of their parents (Bulmer, 1994; Frank, 1985, 1986a). This result is illustrated by the lower surface in Figure 1a. The sex allocation predicted for sister groups (Equation 2) is a constant fraction (1−*f*)/(2(1 + 3*f*)) of that predicted for nonsister groups (Equation 1), independently of the number of co‐foundresses present, and this fraction decreases from ½ to 0 as the degree of inbredness increases from 0 to 1, meaning that the proportional allocation to sons in sister groups is never more than half of what it is in equal‐sized nonsister groups. Note that a female's inbredness is liable to depend on the sizes of groups encountered by her ancestors, such that (unlike for nonsister diplo‐diploid groups) it is likely that sex allocation behaviour predicted for sister groups will depend not only on the local number of foundresses but also the on the distribution of group sizes across the population. In the special case of all groups having the same size, Equation (2) reduces to *z*_D,S_* = (*n* – 1)^2^/(4*n* ^2^) (see Box 1 for details).

Second, we consider a haplo‐diploid (H) mode of inheritance. Here, we find that the unbeatable sex allocation for a female whose group comprises herself and *n*−1 unrelated other females is given by.

(3) $$z_{H,U}^{*}=\frac{\left(n‐1\right)\left(1+f\right)}{2n\left(1+2f\right)},$$

as is illustrated by the upper surface in Figure 1b. Note that, in contrast with the corresponding result given for diplo‐diploidy (Equation 1), the sex allocation exhibited by nonsister groups in haplo‐diploid populations (Equation 3) is dependent upon the degree of inbredness and hence is likely to depend on the distribution of group sizes across the population and not just the size of the focal female's group. In the special case of all groups having the same size, Equation (3) reduces to *z*_H,U_* = ((*n*–1)(2*n*–1))/(*n*(4*n*–1)), as given by Hamilton (1979; see Box 1 for details). More generally, the sex allocation predicted for haplo‐diploid nonsister groups (Equation 3) is a constant fraction (1 + *f*)/(1 + 2*f*) of that predicted for diplo‐diploid nonsister groups (Equation 1), independently of the number of co‐foundresses present, and this fraction decreases from 1 to 2/3 as the degree of inbredness increases from 0 to 1, meaning that it is almost always lower than the corresponding result for diplo‐diploid inheritance.

In contrast, we find that the unbeatable sex allocation for a female whose group comprises herself and *n*‐1 of her sisters is given by.

(4) $$z_{H,S}^{*}=\frac{\left(n‐1\right)\left(1‐f\right)}{8n\left(1+2f\right)},$$

as is illustrated by the lower surface in Figure 1b. This too is dependent upon the degree of inbredness and hence upon the distribution of group sizes across the whole population. In the special case of all groups having the same size, Equation (4) reduces to *z*_H,S_* = (*n*–1)^2^/(2*n*(4*n*–1)). More generally, the sex allocation predicted for sister groups under haplo‐diploidy (Equation 4) is a constant fraction (1−*f*)/(4(1 + *f*)) of that predicted for nonsister groups under haplo‐diploidy (Equation 3), and this fraction decreases from ¼ to 0 as the degree of inbredness increases from 0 to 1, meaning that it is always lower than the corresponding result for nonsister groups.

### 2.1 Comparison of theory and observations

Here we compare model predictions for the haplo‐diploid mode of inheritance with sex ratios observed in *Goniozus*. Abdi et al. (2020) provide data on the sexual composition of 123 broods of offspring produced by *Goniozus nephantidis* (Muesebeck) females held in foundress groups of size ranging from 1 to 8 and with multi‐foundress groups consisting of sister females or nonsister females. The results of null hypothesis significance testing using weighted logistic analysis and the associated equations for the estimated minimal adequate models are presented in Abdi et al. (2020), who also discussed the inclusion or exclusion of a large brood with an outlying sex ratio which was especially influential due to the use of an intrinsically weighted analysis. Here we include the outlier but use unweighted logistic analysis to de‐emphasize the influence of large broods. We obtain the following maximum likelihood estimates of the two empirical relationships between sex ratio, *z*, and foundress number, *n*:

Regression for single foundresses and multiple nonsister foundresses:

(5) $$z\;=1/\left(1+\left(1/\left(\exp\left(0.084n‐1.132\right)\right)\right)\right)$$

Regression for single foundresses and multiple sister foundresses:

(6) $$z=1/\left(1+\left(1/\left(\exp\left(0.071n‐2.072\right)\right)\right)\right)$$

**FIGURE 2:**
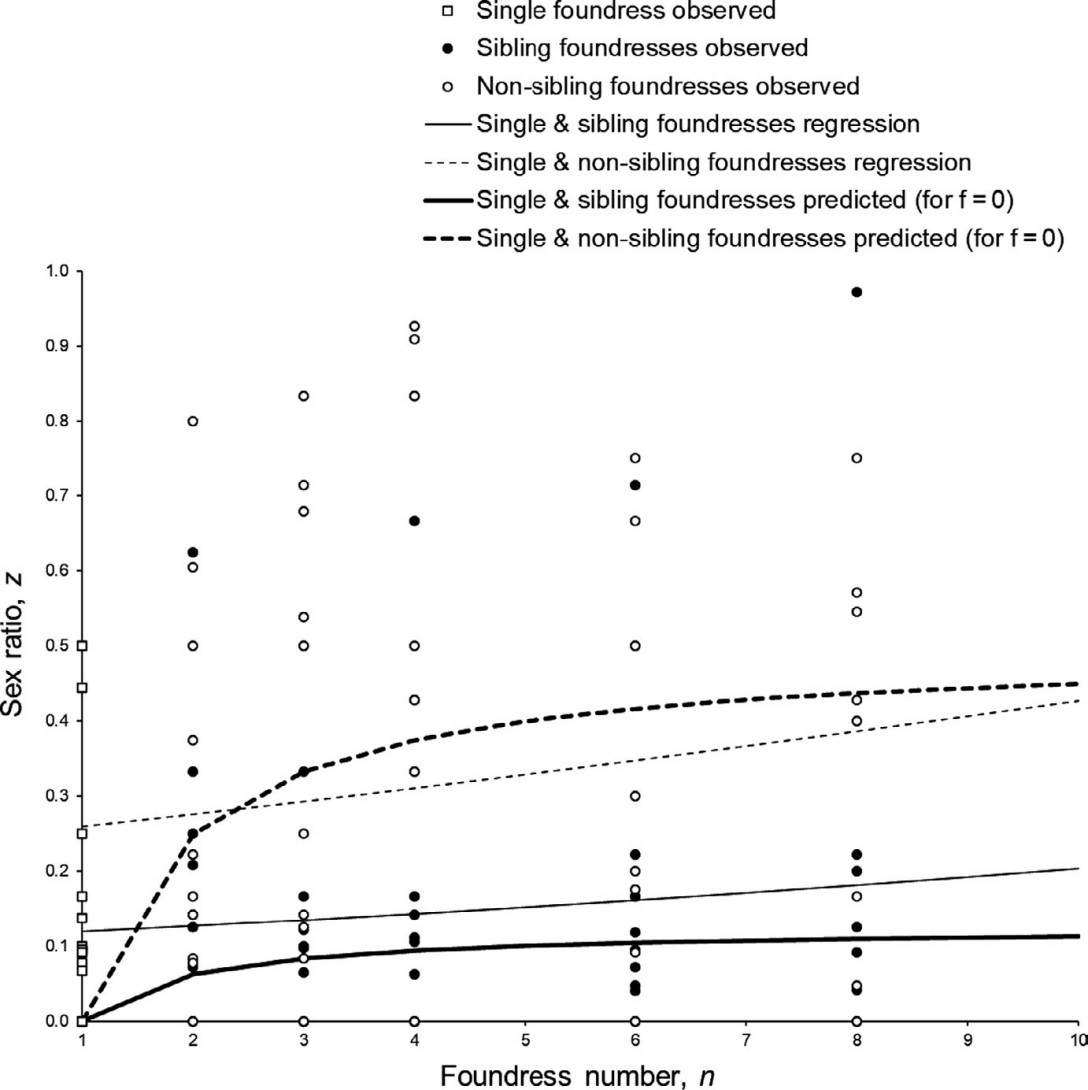
*Goniozus nephantidis* sex ratios: observed and predicted for the haplo‐diploid mode of inheritance. Note that, for the sake of illustration, the prediction lines assume a degree of inbredness *f* = 0, as this is the best‐fitting value, but is likely unrealistic given the life history of *G. nephantidis* (see Discussion for more details)

We explored likely values of *f* (the average inbredness of *G. nephantidis*) by calculating the sums of squared differences between the observed sex ratio of each brood and the sex ratio predicted by Equations 3 and 4 for the given number of foundresses and foundress kinship, across the range of candidate values of *f* (0 to 1): the best‐fit value of *f* was zero. Using *f* = 0, the sex ratios predicted to be produced (Equations 3 and 4) are plotted against *n*, along with the estimated regressions (Equations 5 and 6) and observed brood sex ratio data for *G. nephantidis*, in Figure 2.

We evaluated how well the data fit the model predictions by calculating the sum of squared departures from the observed mean sex ratio (SST) and the sums of squared departures (SSE) from the model for haplo‐diploids (Equations 3 and 4), using data from all replicates, for a range of values of *f*. For *f* = 0, the proportion of variation explained by the model ((SST‐SSE)/SST) was 0.0894. The model provided a better fit to the data than did the overall mean for *f* ≤ 0.35 and for larger values it was worse. As LMC models give notoriously unrealistic predictions for the single foundress case (see below), we also calculated these values with single foundress replicates excluded. The proportion of variation explained was 0.1167 and the model provided a better fit than the mean sex ratio for *f* ≤ 0.5.

Next, we compared the variation explained by Hamilton’s (1979) LMC model for haplo‐diploids (*z*_H,U_* = ((*n*–1)(2*n*–1))/(*n*(4*n*–1))) against the observed mean and found that this model fits the data worse than does the overall mean sex ratio, whether or not single foundress replicates are included. Finally, we calculated the proportion of variation explained by our model for haplo‐diploids (Equations 3 and 4, *f* = 0, Figure 2) compared to Hamilton’s (1979) model (shown plotted along with the same data in fig. 10 of Abdi et al., 2020): across all replicates 23.2% of the variation was explained by including co‐foundress and for multiple‐foundress replicates only this value was 24.44%. We conclude that the inclusion of facultative adjustment according to whether co‐foundresses are sisters or are unrelated females leads to a better match between predicted and observed sex ratios.

## 3 DISCUSSION

We have derived explicit theoretical predictions for sex allocation when females are able to adjust their behaviour according to number and kinship of co‐foundresses, for both diplo‐diploid and haplo‐diploid modes of genetic inheritance. We have shown that females are expected to decrease their allocation of reproductive resources to sons in the presence of both fewer and more closely related co‐foundresses, with the extent of sex ratio bias being dependent upon female inbredness (and hence upon the distribution of group sizes across the population) in all cases except for the classic diplo‐diploid, nonsister‐group scenario considered by Hamilton (1967). These results are in agreement with the more general qualitative predictions of LMC theory (Frank, 1985) and are here rendered in explicit quantitative form for the first time, enabling direct comparison with empirical data.

Our predictions are in line with the experimental data of Abdi et al. (2020) who varied foundress number and relatedness in *Goniozus nephantidis*. Sex ratios of *G. nephantidis* appear to be more affected by co‐foundress relatedness than those of other studied insects and mites: Shuker et al. (2004) report a meta‐analysis across 7 studies, with all effect sizes, *r*, being < 0.3 while, from logistic ANOVA statistics given in Abdi et al. (2020), for *G. nephantidis r* = 0.438. This is most likely associated with the ability of adult *Goniozus* females to discriminate kinship (Abdi et al., 2020; Lizé et al., 2012) which appears lacking in some studied parasitoids (Shuker et al., 2004).

While the development of theory we present has been stimulated by the observed sex ratios of *G. nephantidis* and its predictions match the fitted regressions quite closely, there are several differences between the assumptions of the model and the life history of *Goniozus* that can affect sex ratio. First, the model assumes that local mating groups are sizable, with each foundress contributing a large number, *N*, of offspring. *Goniozus* brood sizes more typically take small integer values which constrains the values of possible brood sex ratios: for the single foundress case, the optimal sex ratio is 1/*N* if daughters are to be able to mate locally (Green et al., 1982) and this especially likely accounts for disparities at *n* = 1 in Figure 2.

Second, the model implicitly assumes that the sexual composition of offspring groups is the same at the time of sex allocation and the time at offspring mating. *Goniozus* broods normally experience some developmental mortality that can alter the sexual composition of broods: this can both select for less biased primary sex ratios than would be predicted in the absence of mortality and also can obscure patterns of sex allocation (Green et al., 1982; Khidr et al., 2013). In the experiment reported by Abdi et al. (2020), offspring mortality was unusually high due to the sustained confinement of multiple‐foundresses and it remains possible that the brood sex ratios observed do not accurately reflect sex allocation decisions made by foundresses.

Third, the model assumes strictly local mating with males dying and only mated females dispersing. In *Goniozus,* males are winged and are observed, in laboratory trials, to disperse from natal broods (Hardy et al., 1999); if nonlocal mating occurs in nature this would select for less female‐biased sex ratios than are predicted by models assuming strict LMC (Nunney & Luck, 1988; West, 2009). This effect is likely masked in Figure 2 on account of the degree of inbredness (*f*) being treated as a free parameter whose value may be adjusted to improve the goodness of fit, with the best fit obtained when females are considered to be completely outbred (*f* = 0). The life history of *Goniozus* suggests that some degree of inbredness (*f* > 0) is expected, which would select for a more female‐biased sex ratio, and therefore compensate for the discrepancy between prediction and observation in the opposite direction arising as a consequence of partial LMC. A more direct estimate of inbredness using molecular markers would be a useful goal for future investigation of the *Goniozus* mating system. Relatedly, the model assumes almost complete dispersal of females following mating and hence negligible competition among related females for reproductive resources. Incorporating limited dispersal of females would be expected to reduce the extent of female bias in sex allocation for both sister and nonsister groups (*cf*. Bulmer, 1986, Frank, 1986b, Taylor, 1988).

A further assumption of the present theoretical model is that females are able to recognize each other as sisters vs nonsisters per se, although the model is agnostic as to whether this involves environmental or genetic kin recognition (Grafen, 1990). A possibility is that females are able to recognize co‐foundresses who eclosed from the same host, without being able to discriminate between those that are actually sisters vs nonsisters (including both more distantly related kin and nonkin co‐foundresses) or that both types of recognition may be used (Lizé et al., 2012, have shown that kin discrimination in one species of *Goniozus* utilizes genetically based and familiarity‐based mechanisms). Discrimination based only on familiarity was considered by Taylor and Crespi (1994) in an analysis of how females are expected to adjust their sex allocation in response to their own dispersal status and that of their co‐foundresses; this instantiated Frank’s (1985) qualitative prediction that closer kinship should result in a more female‐biased sex ratio, though without allowing for the possibility of foundress number variation within populations. On account of familiar females being, on average, less related than confirmed sisters, we would expect adjustment of sex ratios in response to familiarity per se to lead to less female bias than is predicted by the present model.

Finally, a limitation of the present analysis is that it has, following the experimental design of Abdi et al. (2020), focused on the comparison between sister vs nonsister foundress groups and has not explored sex allocation behaviour within groups containing mixtures of sisters and nonsisters (possible for *n* > 2). Mixed groups present a particular mathematical challenge in that they allow for (though do not in all cases necessitate) individuals within a group finding themselves in different circumstances and hence being favoured to exhibit different sex allocation behaviours. When each female's strategy is conditional upon not only her own circumstances but also the circumstances of her co‐foundresses, strategies are required to be solved simultaneously rather than individually. A similar complexity would arise in the presence of partial LMC, whereby the mating success of eclosing males depends upon sex allocation strategies employed globally as well as locally. Such scenarios provide an interesting avenue for future theoretical attention.

## CONFLICT OF INTEREST

Both authors declare that they have no competing interests.

## AUTHOR CONTRIBUTIONS

ICWH identified the problem, AG solved the problem, and both wrote the solution up.

### Peer Review

The peer review history for this article is available at https://publons.com/publon/10.1111/jeb.13719.

## Data Availability

There are no new data associated with this work. The data re‐analysed in this study are available from the Dryad data repository (https://doi.org/10.5061/dryad.905qftth8).
